# Easy, efficient and versatile one-pot synthesis of Janus-type-substituted fullerenols

**DOI:** 10.3762/bjoc.15.87

**Published:** 2019-04-12

**Authors:** Marius Kunkel, Sebastian Polarz

**Affiliations:** 1 University of Konstanz, Universitätsstrasse 10, 78467 Konstanz, Germany

**Keywords:** amphiphile, C_60_Cl_6_, fullerene, fullerenol, one-pot

## Abstract

An efficient one-pot synthesis for Janus-type fullerenol derivatives and how to characterize them is reported. This synthesis provides access to asymmetrically substituted fullerenol with five substituents on one pole of the fullerene and polyhydroxylation moieties, mostly ether and hydroxy groups, on the rest of the fullerene core. As substituents a broad variety of primary amines can be used to obtain Janus-type amphiphilic fullerenols in good to excellent yield. These fullerenol amphiphiles can serve as suitable precursors for further reactions resulting in new applications for fullerenols.

## Introduction

The roman god Janus, who is typically depicted with two faces, metaphorically stands for duality in one person or object. Consequently, nanoparticles characterized by two different hemispheres have been named Janus-type nanoparticles as well, and have attracted major attention due to their special properties [1–2]. The term 'Janus' is much less used in molecular chemistry, presumably because there are not many spherical molecules known. Examples are the giant polyoxometalates reported by Müller et al. [3] and, more importantly for the current paper, fullerene C_60_. Because of the high symmetry of those compounds asymmetric modification is tedious with multiple synthesis and purification steps involved.

Fullerene derivatives are of great interest in numerous research areas such as biological sciences and materials sciences [4–9]. A vast amount of synthetic protocols have been developed over the years to modify fullerenes [10–13]. A particular task was to provide fullerenes with solubility in water. Thus, one important class of fullerene derivatives are the hydroxylated and polyhydroxylated compounds, so called fullerenols (C_60_(OH)*_n_*) [14]. The degree of hydroxylation and with that the solubility of these compounds can be tuned by using different synthetic approaches making it possible to obtain water soluble fullerenols as well as fullerenols that are still soluble in organic solvents [15–19]. The maximum number of OH groups, which could be attached to C_60_ is *n* = 44 [17]. Further derivatizations, where all hydroxy moieties of the compound have been modified, are well known in the literature. These reactions can be achieved by esterification or etherification [20–22]. However, partial modifications are rare, especially when it comes to asymmetric substitutions [23–24]. Although, janus-type fullerenols at which only a part of the fullerene core is hydroxylated are known [25–26], literature lacks fullerenol compounds with Janus-type substitution. The advantage of those special molecular species, e.g., amphiphilic behaviour, was demonstrated in a paper published by our group in 2018 [27]. A fullerenol derivative with a maximum of 21 OH groups on one hemisphere and 5 alkyl chains on the other was reported. The synthesis of this species was elaborate and tedious with relatively low yield, we also failed to introduce more complex substituents than alkyl chains, for instance. The success of the used, multistep synthetic pathway drastically depends on the reactants that are used. One may obtain insoluble or unreactive intermediates, which then prevent the synthesis of the final compound in good yield or to obtain the final compound at all.

For future exploration of the potential of Janus-type fullerenols it is pivotal to establish new synthetic pathways, which allow to introduce a broader variety of substituents and better yield. We report an easy and efficient one-pot approach using C_60_Cl_6_ as a precursor. The attachment of substituents, in our case primary amines, and the polyhydroxylation of the fullerene core are performed simultaneously by using a common phase transfer reaction which enables even the combination of water-soluble substituents with the precursor C_60_Cl_6_. High yields of Janus-fullerenol derivatives bearing five defined substituents on one pole of the C_60_ core combined with in average 19 (+/−3) oxygen containing moieties on the other pole are obtained.

## Results and Discussion

**Scheme 1:**
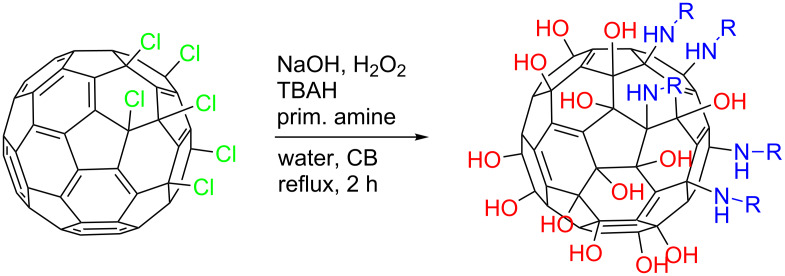
Reaction scheme for the one-pot reaction of C_60_Cl_6_ to produce Janus-type fullerenols (OH)_19+/−3_C_60_(HNR)_5_.

The general procedure for the one-pot preparation of asymmetrically substituted fullerenols is depicted in Scheme 1, and experimental details are presented in the following.

The starting material for all syntheses is C_60_Cl_6_ which was synthesized according to procedure reported by Kuvychko et al. and adapted by our group [27–29]. Though, several compounds are known to undergo the penta-substitution reaction with C_60_Cl_6_, like amines, thiols or alcohols [30–32], it has been observed that primary amines show a high reactivity under these conditions and form stable intermediates during this reaction that can further react. In a general procedure, C_60_Cl_6_ (200 mg, 0.21 mmol) and the primary amine of choice (8 equiv) are dissolved in a chlorobenzene/water mixture (8 mL/40 mL). The mixture is combined with the phase transfer agent tetrabutylammonium hydroxide (0.5 mL of a 30% solution in H_2_O). The reactants for the polyhydroxylation are added, H_2_O_2_ (1.5 mL of a 30% solution) and NaOH (0.7 g). The mixture is heated to reflux until the chlorobenzene phase decolorizes. The reaction is completed after 2 h of reflux. The aqueous phase is separated and poured in methanol to precipitate the crude product. The obtained brown solid is washed with methanol to remove remaining TBAH and NaOH to obtain the sodium salt of the compound. The sodium salt compound can be ion exchanged (amberlite 120) prior to hydrophilic interaction liquid chromatography (silica gel 60, gradient acetonitrile/water 90:10 to 70:30) for purification.

In a first attempt a methyl-protected aminocatechol, namely dimethoxyaniline, was reacted under these conditions. After purification the product was obtained in a good yield of 88%. The compound was characterized as follows.

**Figure 1:**
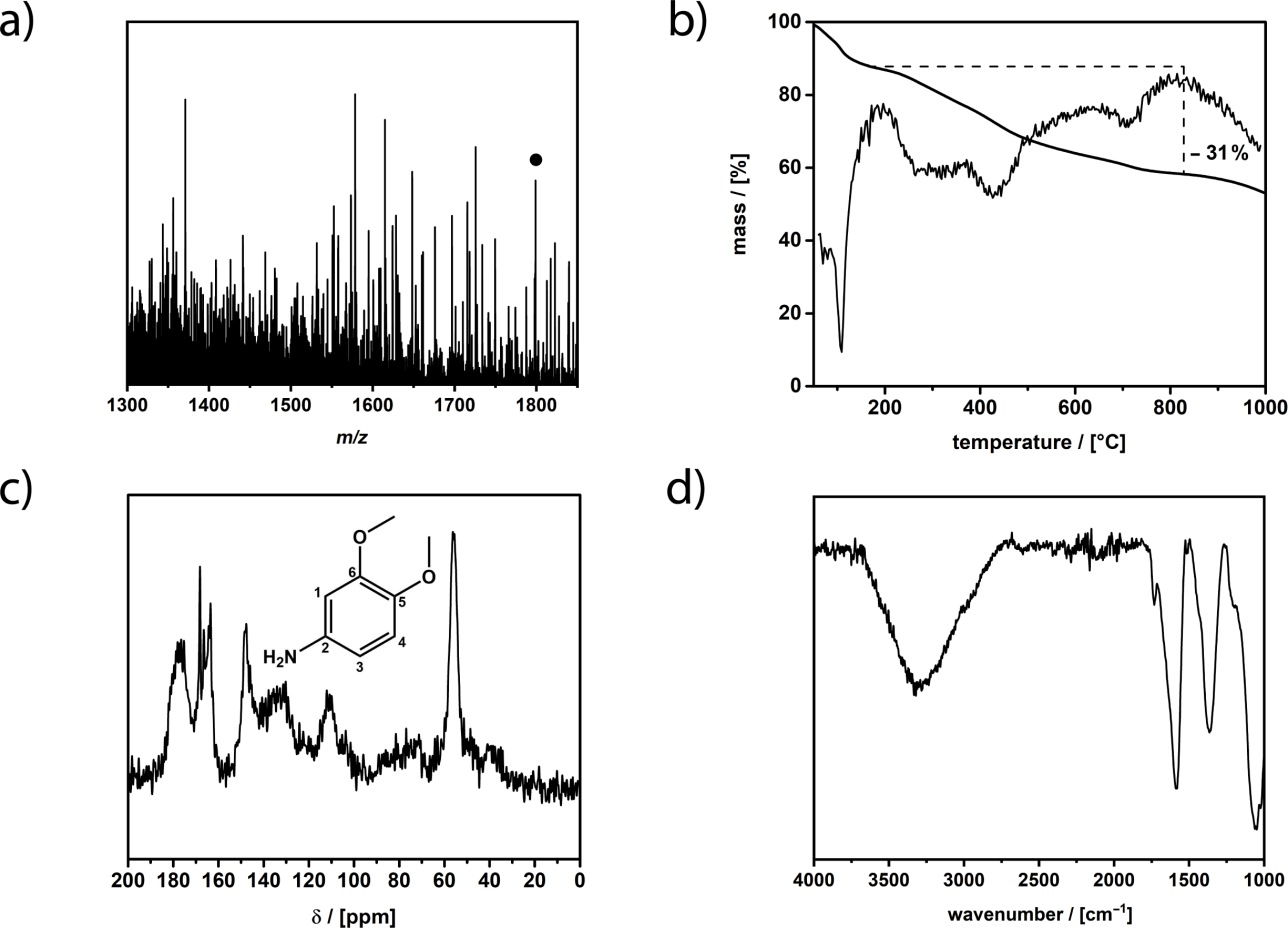
Characterization of fullerenol amphiphile with substituent **1**. a) ESIMS in positive mode, molecular ion peak marked with circle, b) TGA under nitrogen with 5 K/min, c) MAS-NMR (^1^H-^13^C-CP) with structure of substituent, d) ATR–IR.

For the characterization of Janus-type fullerenol amphiphiles more than one method is needed to perfectly identify the compounds. Polyhydroxylation reaction of the fullerene can lead to a mixture of several oxygen moieties like hydroxy groups, diols, ketones, hemiketals, epoxides and ethers. The general formula of the compound is identified by electrospray ionization mass spectrometry (ESIMS). The degree of polyhydroxylation and the number of substituents is determined and confirmed with thermogravimetric analysis (TGA) and the nature of oxygen moieties as well as the substituents attached are evaluated via a ^13^C magic angle spinning nuclear magnetic resonance spectroscopy (MAS NMR) ^1^H-^13^C-CP experiment. An overall information of the compound is obtained by attenuated total reflection infrared spectroscopy (ATR-IR). Figure 1 shows the results of the characterization exemplarily for the fullerenol amphiphile with dimethoxyaniline as substituent (all characterization data can be found in Supporting Information File 1).

ESIMS (Figure 1a) shows a rather complex fragmentation pattern similar to unsubstituted fullerenol compounds known in the literature [33]. The general complexity of the spectrum derives from the isomerization and fragmentation of the oxygen species in addition to the fragmentation of the substituents. The signals can be assigned with a general formula [M − *x*H_2_O − *y*H − *z*O − *v*(HNR)]^a−/+^. The molecular ion peak of the janus-fullerenol with **1** as substituent can be identified at *m*/*z* 1798.6 (1798.6) which corresponds to (H)_13_(O)_19_C_60_(HNR)_5_^+^ and with that fits 19 oxygen species. As already mentioned there are several oxygen species present in the compound which are further identified later on with MAS NMR. The formula of the compound indicates that there are 12 hydroxy groups present and 7 other oxygen species. The TGA (Figure 1b) is another method to confirm the number of attached oxygen species and substituents. The first step up to 180 °C fits the release of secondary and tertiary bond water. The measurement shows that 12 H_2_O molecules are bound to the fullerene. The second step up to around 800 °C can be assigned to the release of the different oxygen species, which also explains the little steps which correspond to different oxygen types. In addition to the oxygen species attached to the fullerene core, the methoxy moieties of the substituent are also released. The average number of oxygen species can be calculated to 19.5, which fits the data obtained from ESIMS. Above 800 °C the decomposition of the core structure and the substituents starts. After that step only amorphous carbon remains and the compound is completely decomposed. Finally, the MAS-NMR (Figure 1c) confirms the attachment of the substituents and that the structure of substituents is unchanged. Furthermore, it provides evidence to determine the kind of the oxygen species [34–35]. At 175 ppm the signals of C=C–O groups are located. These signals are rather intense which gives evidence that the most prominent oxygen structure motive besides hydroxy groups are ethers. This may result from the reaction conditions since it was already shown that the reaction conditions influence the obtained oxygen moieties. The signal at 163 ppm can be assigned to carbon atoms 5 and 6 of the substituent. Carbon atom 2 of the substituent is located at 148 ppm. Remaining sp^2^-hybridized carbons of the fullerene core are located between 142 and 120 ppm. At 110 ppm the remaining carbons of the substituent are located. The sp^3^-hybridized carbons of the fullerene core at which the hydroxy groups are attached are located between 80 and 60 ppm. Finally, the carbon atoms of the methoxy moieties can be found at 55 ppm. Figure 1d displays the ATR–IR spectrum of the compound which confirms the results from the other analytical methods. Most prominent in the spectrum are the signals of the polyhydroxylation moieties. Signals at 3293, 1579, 1358, 1200 and 1049 cm^−1^ can be assigned to the O–H, C–O–C, C–O and C–OH vibrations.

The scope of the reaction was further tested with other primary amines, aliphatic as well as aromatic (Table 1). The aliphatic amines **2** and **3** react in good yields over 70%. For these compounds the reaction needs about 2 h to be finished. The reaction with compound **2** leads to a little amount of insoluble byproduct which might be double reacted amine although an excess of amine was used. The loss of yield for the reaction with **3** results mainly from byproducts that are not penta-substituted. The aromatic compound **4** reacts the fastest and the reaction was completed after 20 min. Strong foaming indicates the completion of the reaction. Almost no byproduct could be isolated here.

**Table 1:** Scope of the reaction and isolated yields.

Entry	Reactant	Yield^a^

1	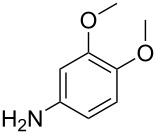 **1**	88%
2	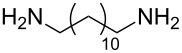 **2**	70%
3	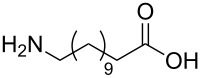 **3**	75%
4	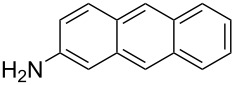 **4**	90%
5	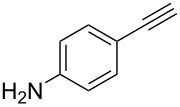 **5**	82%
6	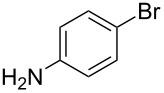 **6**	80%

^a^Yield after purification.

Of special interest for this reaction are compounds with additional functional groups like alkynes or bromides that can be reacted with other compounds in further reactions, e.g., click reactions or Sonogashira coupling. Systems for such reactions are tested with compounds **5** and **6**. These compounds react in high yields up to 90%. The additional functional groups, which must of course be not base-labile survive the reaction conditions with no harm. The attached moieties, no matter if electron withdrawing or donating, do not seem to influence the reaction. Moreover, neither the degree of polyhydroxylation nor the kind of attached oxygen species seem to be influenced by the attached substituents. For all compounds the average degree of polyhydroxylation is 19(+/−3). Noteworthy, neither the degree of polyhydroxylation could be increased nor the nature of oxygen species could be varied by extending the reaction time.

## Conclusion

In summary, we have established a new and easy one-pot method for the synthesis of Janus-type fullerenol amphiphiles. The reaction includes a wide range of primary amines reaching from aliphatic amines over aromatic amines to further functionalized amines. All resulting compounds have the general formula (OH)_19+/−3_C_60_(HNR)_5_, whereas, mainly hydroxy and ether moieties are included. They have been characterized with ESIMS, TGA and MAS NMR. These compounds can serve as precursors for further modified fullerenols or as true surfactants.

## Supporting Information

File 1 General methods and characterization data.
