# Supplementary material for: Easy, efficient and versatile one-pot synthesis of Janus-type-substituted fullerenols
Source: Beilstein J Org Chem. 2019 Apr 12;15:901–5. doi: 10.3762/bjoc.15.87 (PMC6466692; doi:10.3762/bjoc.15.87)
Supplement: File 1 — General methods and characterization data. [file Beilstein_J_Org_Chem-15-901-s001.pdf]

BEILSTEIN JOURNAL OF ORGANIC CHEMISTRY

Supporting Information

for

Easy, efficient and versatile one-pot synthesis of Janus-type-substituted fullerenols

Marius Kunkel and Sebastian Polarz

Beilstein J. Org. Chem. **2019,** *15,* 901–905. doi:10.3762/bjoc.15.87

General methods and characterization data

License and Terms: This is a supporting information file under the terms of the Creative Commons Attribution License (http://creativecommons.org/licenses/by/4.0). Please note that the reuse, redistribution and reproduction in particular requires that the authors and source are credited.
The license is subject to the *Beilstein Journal of Organic Chemistry* terms and conditions: (https://www.beilstein-journals.org/bjoc)

General methods

Synthesis that acquired inert gas atmosphere were performed using general Schlenk techniques under argon atmosphere. The solvents were dried according to standard literature and stored under argon. Water was deionized with Millipore Milli-Q. All starting materials used for the syntheses were purchased from commercial sources unless stated differently. The fullerene C_{60} (pur. 99.9%) was purchased from Research & Production Company "Modern Synthesis Technology".

Analytical methods

Mass spectra were measured on Bruker amazon SL in pos. or neg. mode via direct inject from a methanolic solution. For assignment of signals work of Silion, Mihaela, et al. was used as reference.[1] Assignment was done as follows: $[M - xH_2O - yH - zO - v(HNR)]^{a-/+}$. The molecular ion peak was identified in combination with results from TGA and NMR. TGA was measured on Netzsch Jupiter STA 449 F3. All measurements were performed under nitrogen atmosphere with 80 mL/min flowrate and with a heating rate of 5 K/min. NMR measurements were performed on Bruker Avance II 400 solid-state NMR at 295 or 350 K and with 10 kHz rotational speed. Number of scans 5k–10k with D1 = 10 to 100 sec. Attenuated total reflection−infrared (ATR−IR) spectra were measured with a Perkin Elmer100 Spectrum spectrometer including an ATR unit.

Characterization data

Entry 1

Substituent

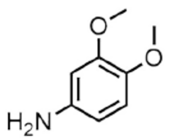

ESIMS

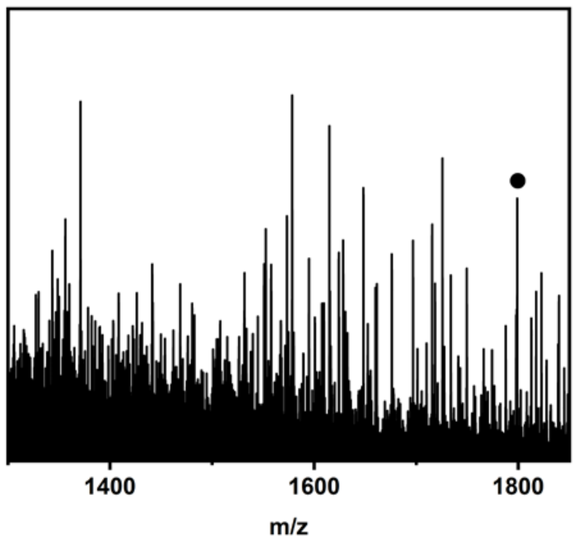

$[H_{13}O_{19}C_{60}(NC_8H_{10}O_2)]^+$ *m/z* = 1798.7 (1798.6).

MAS NMR

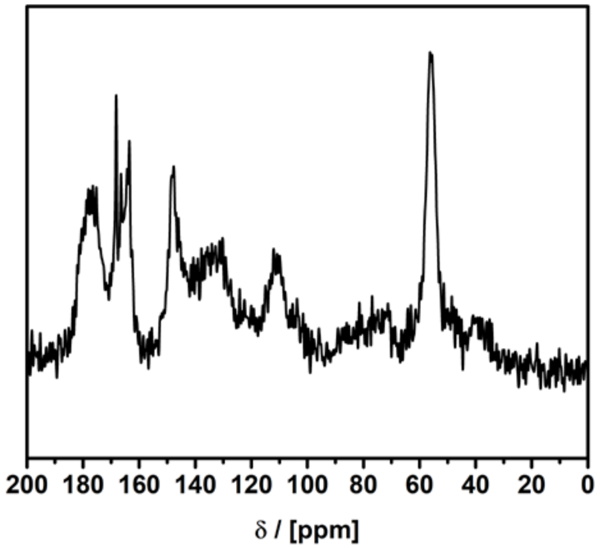

^1H-^{13}C-CP MAS-NMR (100 MHz): δ (ppm): 175 C=C–O, 163 160 C(sp^2)-N, 148 ppm C sp^2, 142–120 ppm C sp^2, 110 ppm C sp^2, 80–60 ppm C-OH, 55 ppm O–CH_3.

TGA

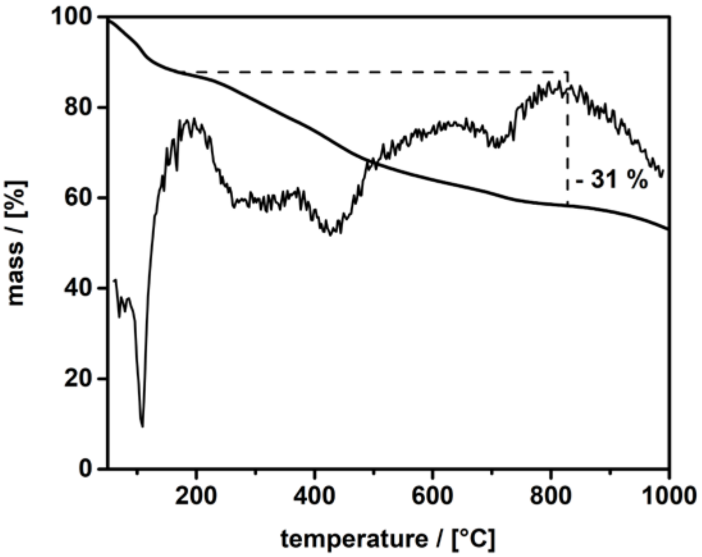

1. Step 50 °C – 180 °C 13%
2. 180 °C – 800 °C 31% → 19.5 oxygen species
3. >800 °C 56%

ATR-IR

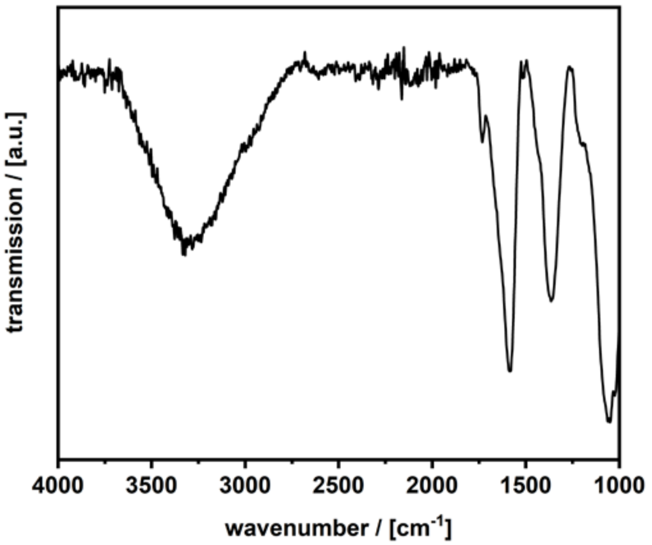

IR (powder): 3293, 3000, 2971, 1579, 1440, 1358, 1200, 1049 cm^{-1}.

Entry 2

Substituent

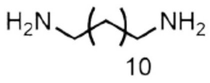

<u>ESIMS</u>

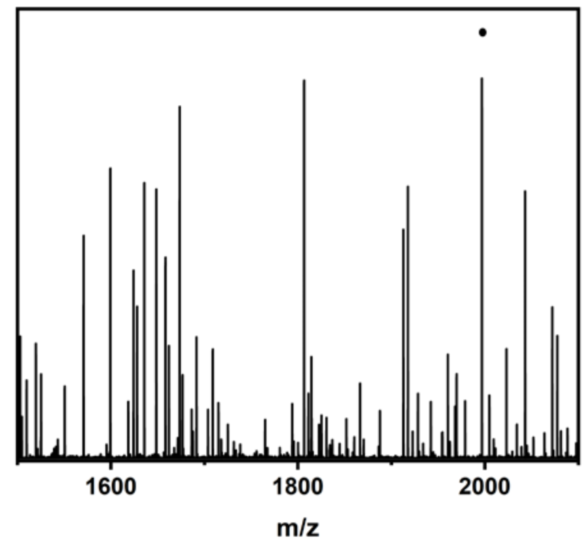

$[H_7O_{17}C_{60}(N_2C_{12}H_{27})]^-$ *m/z* = 1996.9 (1996.5).

<u>MAS-NMR</u>

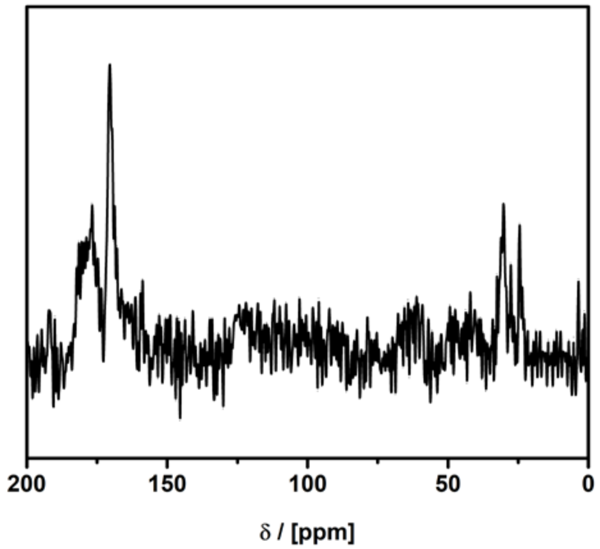

^{1}H-^{13}C-CP MAS-NMR (100 MHz): δ (ppm) 177 C=C–O, 150 C sp^2 (fullerene), 60–40 C–OH, 42 C–N-, 29 C sp^3 (chain).

TGA

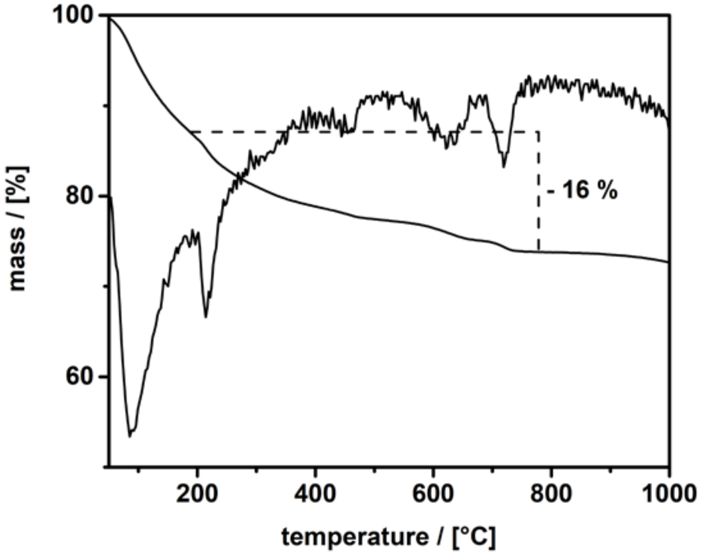

1. Step 50 °C – 190 °C 13%
2. 190 °C – 775 °C 16% → 18 oxygen species + amine endgroups
3. >775 °C 71%

ATR-IR

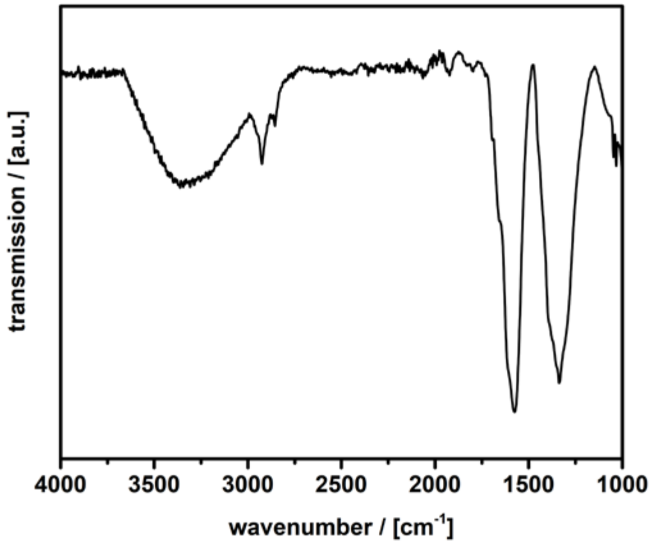

IR (powder): 3320, 2923, 2852, 1655, 1574, 1393, 1327 cm^{-1}.

Entry 3

Substituent

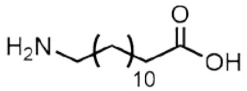

ESIMS

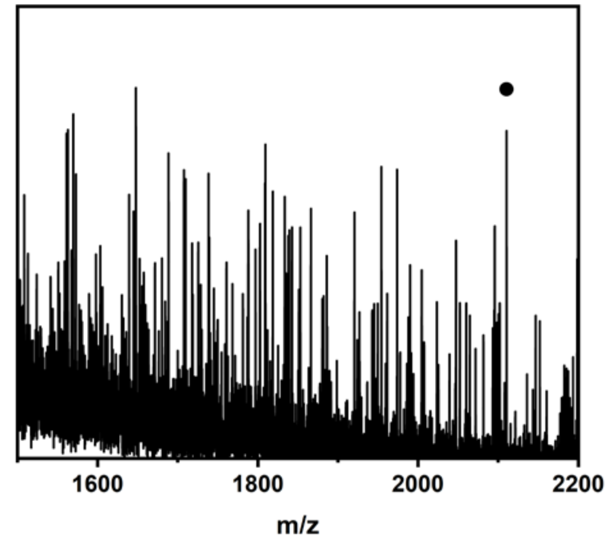

$[H_{14}O_{19}C_{60}(NO_2C_{12}H_{24})]^+$ *m/z* = 2110.2 (2110.4).

MAS-NMR

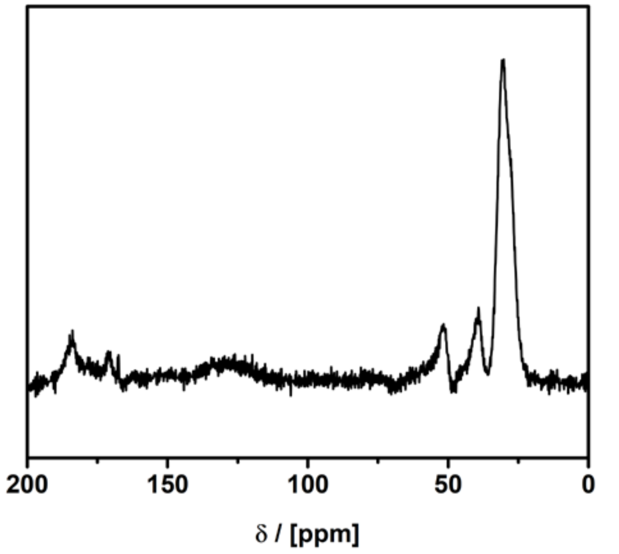

^1H-^{13}C-CP MAS-NMR (100 MHz): δ (ppm) 184 COOH, 170 C=C-O, 160 C sp^2 (fullerene), 75–50 C–OH, 51 C–N, 39 C–COOH, 29 C sp^3 (chain).

TGA

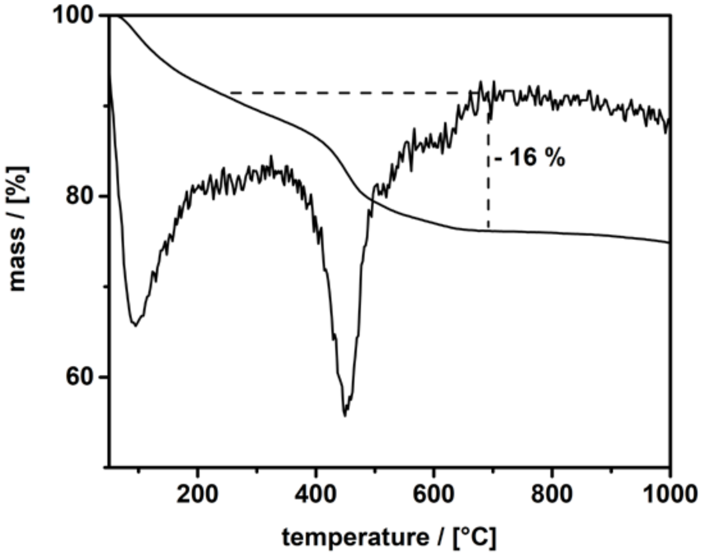

1. Step 50 °C – 190 °C   9%
2. 190 °C – 775 °C   16% → 21 oxygen species
3. >775 °C   75%

ATR-IR

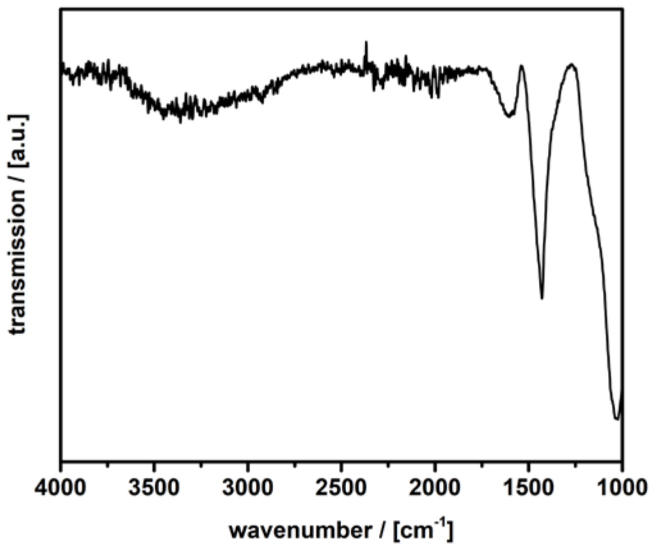

IR (powder): 3340, 2923, 2841, 2300, 1599, 1431, 1158, 1028 cm^{-1}.

Entry 4

Substituent

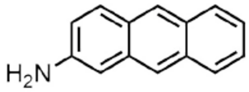

<u>ESIMS</u>

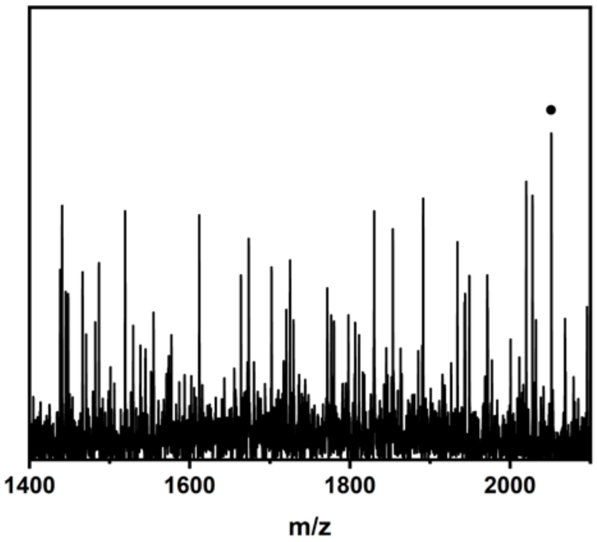

$[H_{17}O_{21}C_{60}(NC_{14}H_{10})]^-$ *m/z* = 2051.1 (2051.0).

<u>MAS NMR</u>

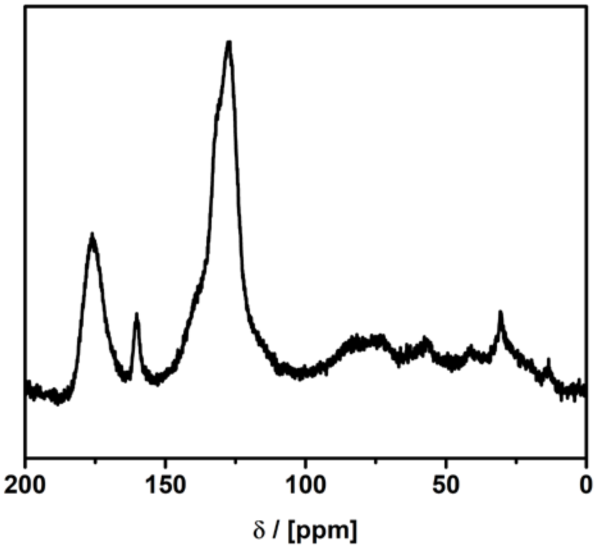

^{1}H-^{13}C-CP MAS-NMR (100 MHz): δ (ppm) 175 C=C-O, 160 C(sp^2)-N, 127 C sp^2, 75–50 C–OH, 29 SSB.

TGA

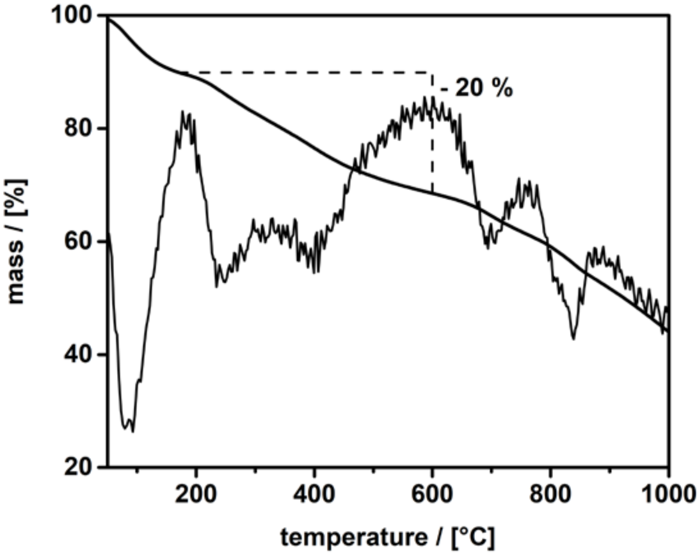

1. Step 50 °C – 180 °C 12%
2. 180 °C – 600 °C 20% → 22 oxygen species
3. >600 °C 68%

ATR-IR

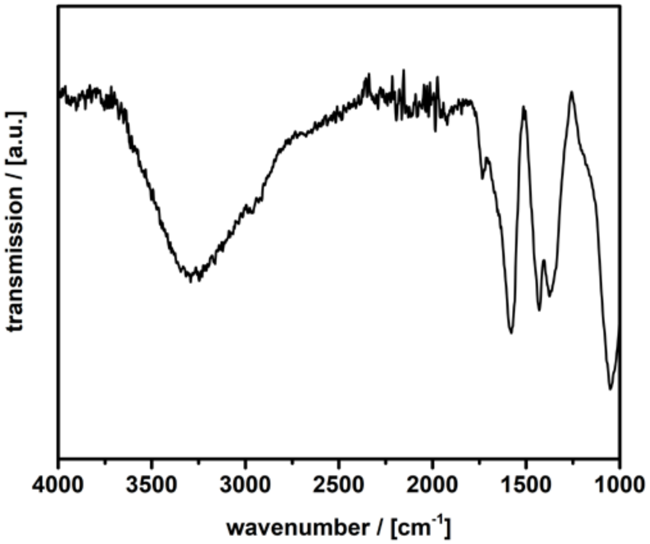

IR (powder): 3270, 2970, 2910, 1732, 1577, 1433, 1361, 1058 cm^{-1}.

Entry 5

Substituent

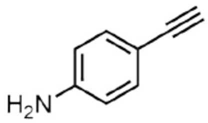

ESIMS

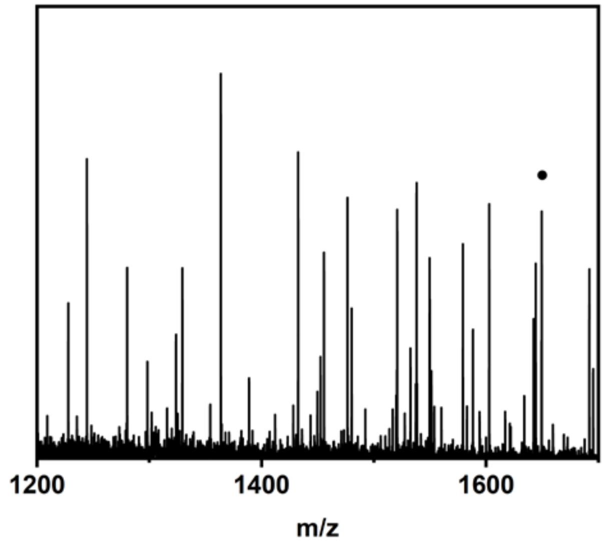

$[H_{12}O_{21}C_{60}(NC_8H_6)]^-$ *m/z* = 1649.5 (1649.5).

MAS-NMR

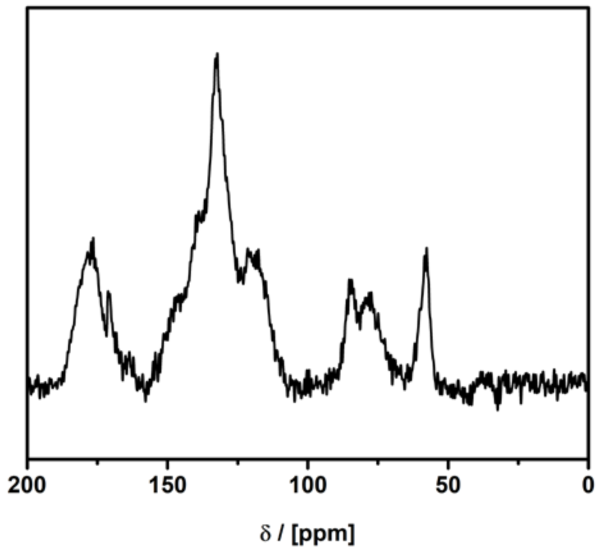

^1H-^{13}C-CP MAS-NMR (100 MHz): δ (ppm) 175 C=C-O, 132 C sp^2, 120 C sp^2, 84 C sp, 77 C sp, 60–40 C–OH.

TGA

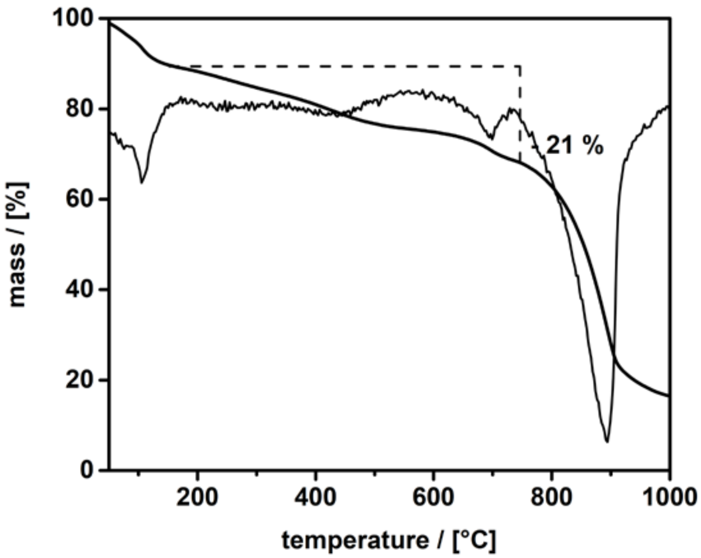

1. Step 50 °C – 150 °C 11%
2. 190 °C – 750 °C 21% → 22 oxygen species
3. >750 °C 68%

ATR-IR

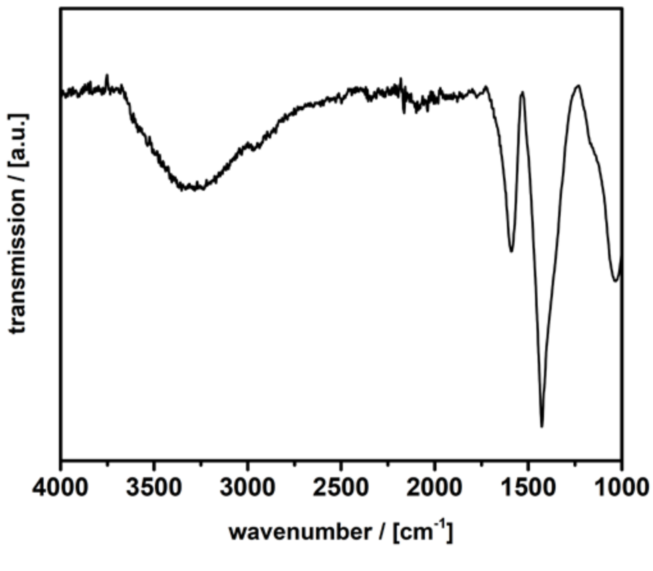

IR (powder): 3280, 2983, 2926, 1593, 1422, 1166, 1033 cm^{-1}.

Entry 6

Substituent

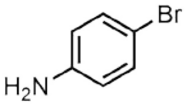

ESIMS

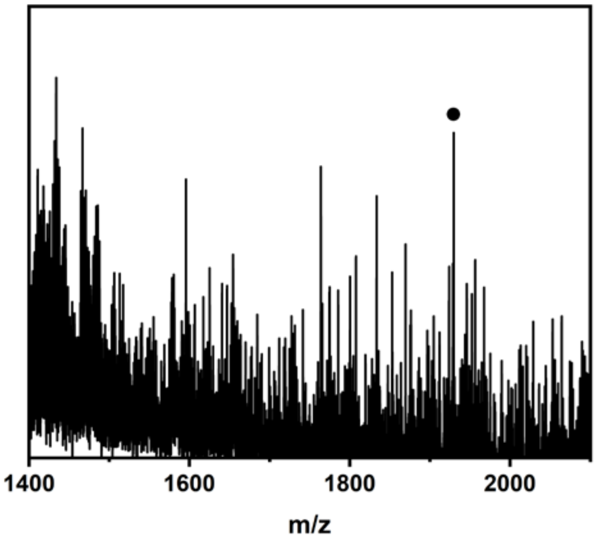

$[H_{18}O_{21}C_{60}(NC_8H_5Br)_5]^+$ *m*/*z* = 1929.7 (1929.9)

MAS-NMR

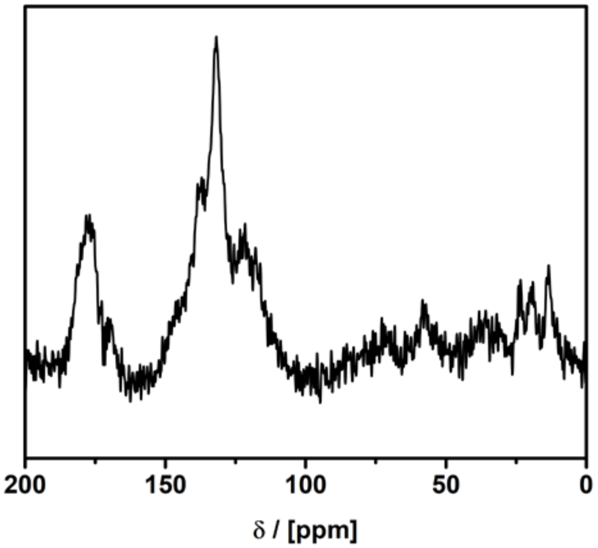

^1H-^{13}C-CP MAS-NMR (100 MHz): δ (ppm) 175 C=C–O, 140 C(sp^2)-N, 130 C sp^2, 118 C sp^2, 65–40 C-OH, 17 SSB.

TGA

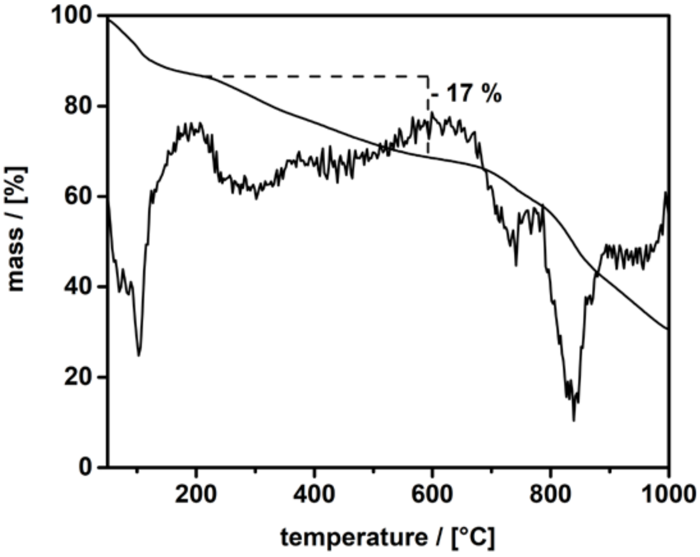

1. Step 50 °C – 200 °C 15%
2. 200 °C – 600 °C 17% → 21.5 oxygen species
3. >600 °C 68%

ATR-IR

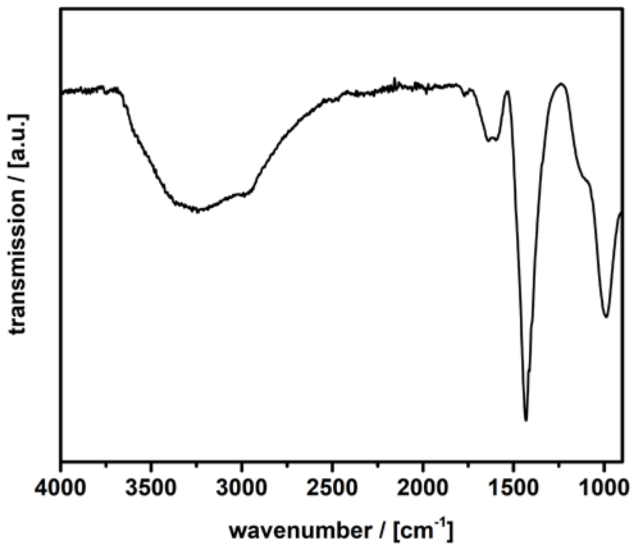

IR (powder): 3240, 2982, 2942, 1639, 1588, 1429, 1117 cm^{-1}.

References

1. Silion, M.; Dascalu, A.; Pinteala, M.; Simionescu, B. C.; Ungurenasu, C., A study on electrospray mass spectrometry of fullerenol C60 (OH) 24. *Beilstein J. Org. Chem.* **2013,** *9*, 1285.
